# Cessation of deliberate self harm following eye movement desensitisation and reprocessing: A case report

**DOI:** 10.1186/1757-1626-1-177

**Published:** 2008-09-23

**Authors:** Derek F McLaughlin, Iain W McGowan, Michael C Paterson, Paul W Miller

**Affiliations:** 1 University of Ulster, Shore Road, Newtonabbey, Co Antrim, Northern Ireland; 2 University of Ulster, Northlands Road, Londonderry, Northern Ireland; 3 TMR Health Professionals, Newforge Lane, Belfast, Northern Ireland

## Abstract

We present a case report of an eighteen year old female patient presenting with a psychological trauma related complaint. Part of the manifestation of the complaint included acts of self cutting over a number of years. Following two sessions of Eye Movement Desensitization & Reprocessing with one of the authors (DM) her self cutting ceased. This is maintained at thirteen months follow up. We conclude that Eye Movement Desensitization & Reprocessing may be an effective treatment option in reducing repeat self harm where traumatic events are noted to be the precursor to deliberate self harm.

## Background

Deliberate Self-Harm (DSH) is a major public health issue across the developed world. It is suggested that 170000 presentations are made to Accident & Emergency Departments annually in the United Kingdom at an estimated economic cost over €40 million. This, coupled with the established relationship between DSH and completed suicide, has led to policy initiatives in the US, Canada and the devolved administrations in the UK seeking to address the topic [1-3].

Current literature on the effectiveness of interventions have a number of limitations. A recent systematic review of psychological and pharmacological interventions found major methodological limitations, primarily a small sample size and lack of adequate power to detect meaningful treatment effects [4].

It has been suggested that psychological trauma is a risk factor for engagement in DSH. This paper reports a case of an 18 year old female engaging in DSH referred for a psychological related trauma problem. She was treated using Eye Movement Desensitization and Reprocessing (EMDR).

EMDR is a non-pharmacological treatment for psychological trauma. Randomized controlled trials have shown its efficacy with a number of groups including a reduction of Post Traumatic Stress Disorder (PTSD) symptoms in war veterans[5], survivors of sexual abuse[6] and conduct disorders in young males[7] (further references are available at ). It has been recommended as an effective treatment for PTSD in adults by NICE (2005), as well as the Dept of Defense and Veterans Affairs in the USA.

As with any psychological therapy, the exact mechanisms involved during EMDR are unknown. However, the Adaptive Information Processing Model [8] attempts to explain what has been observed clinically. It is thought that an original 'traumatic' incident becomes locked in the central nervous system in state-specific form, being held in a memory network. Such memories can have a lasting negative impact on a person's life. In response to triggers in the present day, the traumatic memory is activated and the client re-experiences aspects of the original event. These can be visual, auditory, olfactory or somatic sensations, emotions, and/or negative self thoughts. The use of alternate (bilateral) stimulation of each brain hemisphere, whilst noticing aspects of the traumatic memory, appears to aid the brain to process the original incident, moving to adaptive resolution as with the physical healing process. Clients often report that the event is "in the past" and no longer disturbing to them.

Alternate or bilateral stimulation can be achieved a number of ways; for example by having the client follow the therapist's finger with their eyes keeping their head still (hence the 'eye movement' in EMDR), alternate hand tapping, or alternate audio tones such as clicking or snapping fingers on each side of the client's head so that auditory stimulation is through the left and right ears in turn.

**Figure 1:**
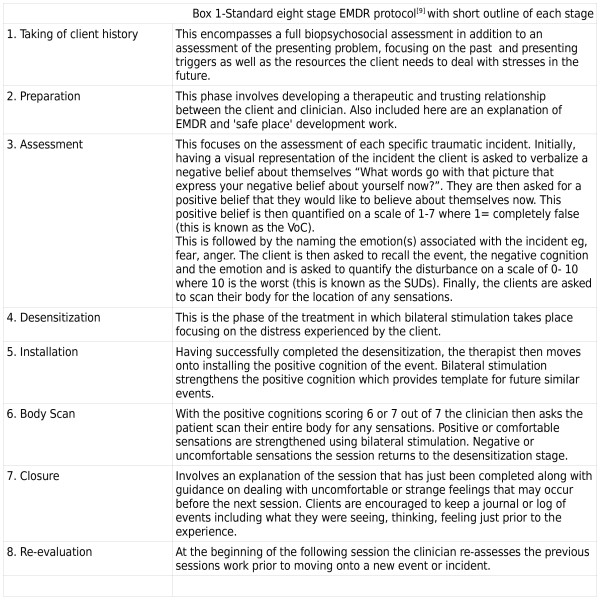
EMDR protocol.

The standard eight phase protocol for EMDR[8] (see Figure 1) was used in this case presentation. This incorporates a three-pronged protocol dealing with past, then present, then future; i.e. addressing the original incident, then current triggers that cause the maladaptive behaviour, and lastly installing a desirable response increasing self efficacy.

## Case presentation

The patient, Natalie, is an eighteen year old single female.

Natalie self referred to a private mental health out-patient service specializing in the treatment of psychological trauma in January 2007. She reported labile mood that often resulted in a transient low mood accompanied by anger. During these transient periods Natalie would engage in DSH; typically cutting herself on her inner thighs.

Natalie reported that, on a descriptive scale of 0 – 10 (where 0 is no anger and 10 is intense anger) that she would cut her self when her anger level reached 8. She reported a number of triggers for her anger, but that these triggers would always relate in some way to events from her past that she remained very angry about. In essence, the presenting event combined with her past life events led to her anger rising to the point where she would cut to relieve herself of this distress.

During the initial assessment Natalie reported an uneventful childhood in relation to her emotional and physical health, her school life or family life. She is the eldest of three children and is currently studying at University.

In the fourth year of post-primary education, around the age of 14 years Natalie reports that her best friend "turned on her". This led to a prolonged period of bullying by this girl on Natalie. Subsequently, and as a direct result of this bullying, Natalie reports becoming depressed and starting to engage in cutting herself. She also reported one incident of tying a scarf around her neck.

Following this incident Natalie was referred to her General Practitioner who prescribed Amitryptaline and made arrangements for her to be seen by her local Child and Adolescent Mental Health Services (CAMHS).

She reports that the input from the CAMHS service was initially beneficial and that their input coupled with the antidepressant medication resulted in an improvement in her mood and a cessation of self cutting.

Aged 16 years, Natalie embarked upon a relationship for two years that, on reflection, she now reports was "unhealthy". She states for example, that her alcohol consumption was a times around 80 units per week. This relationship was also characterized by humiliation and bullying of Natalie. She ended the relationship when she was 18 years of age.

Also, at age 18 years Natalie was discharged from the CAMHS services and no further referral to adult services were made. Natalie stopped taking her medication and started to resume cutting herself as a reaction to the bullying she had experienced during her adolescent years.

## Intervention

Natalie was seen a total of seven times over a five month period in early 2007 by one of the authors (DM). During the initial interview, a full bio-psycho-social assessment was undertaken during which trigger factors for her self harming and the impact of the bullying were explored. The second, third and fourth meetings explored in further depth the triggering factors for the self harming behaviour.

The standard protocol for EMDR [8] was then started, from the fifth meeting onward, initially with safe place development. The 'anchoring' of the safe place and the bi-lateral simulation were achieved by using alternate finger clicks. A 'safe place' is an imaginary place the client can go to activate the parasympathetic nervous system, either following treatment, between sessions or during EMDR sessions when they need time-out due to exhaustion or personal choice.

During the sixth session Natalie was asked to identify an event from her past that still caused her anger. Rated on a scale of 0 – 10 Subjective Units of Distress (SUDs) (where 10 is the worst), she gave this incident a score of 6/10. Natalie was then asked to state positively how she would like to feel or recall this event. Such positive cognitions can include statements such as "I am safe", I am a good person" and "I can trust my judgment". She rated her positive cognition on the incident as 0/7 (where 0 is completely false and seven completely true). Bilateral stimulation was then administered and the distress experienced by Natalie quickly reduced to 0/10.

Having reduced the subjective distress, the treating author then moved to address the desired positive cognition. Again, bilateral stimulation with alternate clicks was used. The validity of the desired cognition (VoC) quickly increased to 7/7.

In her seventh and final contact with the authors the same procedure as the six session was followed. On this occasion, Natalie identified an event that caused distress at a level of 7/10. Again, this quickly reduced to 0/10 during the course of the alternate finger clicks. Likewise the VoC that she Natalie wanted associated with this event increased to 7/7.

### Evaluation

Returning after the first session in which the assessment took place Natalie reported a single incident of cutting herself. Of the remaining contact two sessions were employed to use EMDR to tackle the root cause of Natalie's anger and therefore her DSH. For the initial drafting of this manuscript Natalie was contacted by one of the authors (DM) for a follow up interview at thirteen months post discharge. She reported that she had had no further episodes of DSH despite a number of challenging events in her life.

## Conclusion

Trauma and traumatic events have long been associated with deliberate self harm [9]. In this case report we show the effectiveness of an approach that resolves the underlying root causes of the self harming behaviour, subsequently stopping the maladaptive behaviour whilst at the same time giving Natalie the resources to use adaptive coping strategies when feeling threatened.

This appears to be the first report of EMDR having a positive effect in stopping self-injurious behaviour. Whilst trauma and traumatic events are recognised as precipitant factors in self harming acts no published research on the secondary effectiveness of trauma interventions are apparent.

We suggest that EMDR may be an effective treatment option in reducing acts of self harm where traumatic events have been identified as a factor in the behaviour.

## Competing interests

All four authors have completed EMDR training.

PM & MP are Consultant EMDR Practitioners.

MP is President – Elect of EMDR Association UK and Ireland.

## Authors' contributions

The paper was conceived and, initially, drafted by DM & IM. PM, MP & DM extracted and initially anonymized the data from the case notes. IM analyzed the data and all four authors were involved in the interpretation of the data. All four authors contributed to the writing of the manuscript.

## Consent

Written informed consent was obtained from the patient for publication of this case report and accompanying images. A copy of the written consent is available for review by the Editor-in-Chief of this journal. Natalie suggested the authors use her true name rather than a pseudonym.

## Patients perspective

Natalie was offered the opportunity to contribute to the paper from her perspective. She declined the offer, however in a separate e-mail to one the authors she thanked him and stated;

" [I] never thought I would ever reach the stage of being completely happy with myself! Took some work, but got there in the end".
